# Ring-Opening Polymerization (ROP) and Catalytic Rearrangement as a Way to Obtain Siloxane Mono- and Telechelics, as Well as Well-Organized Branching Centers: History and Prospects

**DOI:** 10.3390/polym14122408

**Published:** 2022-06-14

**Authors:** Kseniya A. Bezlepkina, Sergey A. Milenin, Natalia G. Vasilenko, Aziz M. Muzafarov

**Affiliations:** Enikolopov Institute of Synthetic Polymeric Materials, Russian Academy of Sciences, 117393 Moscow, Russia; ksuhenyaus@gmail.com (K.A.B.); n-vasilenko@mail.ru (N.G.V.)

**Keywords:** polysiloxanes, catalytic rearrangement, ring-opening polymerization, polydiorganosiloxane telechelics

## Abstract

PDMS telechelics are important both in industry and in academic research. They are used both in the free state and as part of copolymers and cross-linked materials. At present, the most important, practically used, and well-studied method for the preparation of such PDMS is diorganosiloxane ring-opening polymerization (ROP) in the presence of nucleophilic or electrophilic initiators. In our brief review, we reviewed the current advances in the field of obtaining polydiorganosiloxane telechelics and monofunctional PDMS, as well as well-organized branching centers by the ROP mechanism and catalytic rearrangement, one of the first and most important reactions in the polymer chemistry of silicones, which remains so at the present time.

## 1. Introduction

Polyorganosiloxanes are one of the most important classes of polymers with great practical importance. The nature of the backbone determines the set of unique characteristics of these macromolecular compounds, making them indispensable in the creation of materials widely used in various fields of practice, ranging from construction, engineering, agriculture, and environmental protection to medicine, cosmetics, pharmaceuticals, and home care products [1,2,3,4,5]. Siloxanes have low surface tension, hydrophobicity, good surface wettability, damping properties, low glass transition temperature and frost resistance, low temperature dependence of physical properties, low toxicity and flammability, and they are safe for the environment [6,7,8]. The most important property of siloxanes is biocompatibility, which are already actively used in science and technology [9]. The development of new high-tech industries, such as organic electronics and photonics, 3D printing, gas separation, and drug delivery, has led to a further expansion of the applications for various types of organosiloxane polymers [8,9,10,11,12,13,14].

The significant feature of organosiloxane polymers in comparison with classical organic macromolecules is determined by a significantly higher Si–O bond energy than the C–C and C–O bonds in the main chains of organic polymers [15]; it determines high-temperature characteristics, and slight changes in physical properties over a wide temperature range. A longer bonds and wider angle of rotation compared to carbon analogues determine the high flexibility of the siloxane chain, low glass transition temperature, and high gas permeability. A weak intermolecular interaction also determines one of the significant disadvantages of organosiloxanes: their relatively low physical and mechanical properties [16]. Searching for ways to overcome this significant drawback, while maintaining the main advantages of this class of polymers, has continued throughout the history of their development.

More recently, however, these researches have reached the highest level of property control—the structure of synthesized polymers is improving. As a result, many highly organized polymeric structures have appeared, among which dendrimers, multi-arm stars, and molecular brushes can be noted [17,18]. These macromolecular systems have a number of unique properties that were first made available through the use of well-organized functional precursors. The appearance of dense molecular brushes in which polymer side chains were grafted to linear skeletons [19] marked other forms of intermolecular interaction. In particular, it facilitated the disentanglement of chains in polymer melts [20,21,22] and opened up prospects for creating a brush-like architecture for the creation of modern elastomers [23], thermoplastics [24,25], and molecular ensembles [26,27]. In contrast to linear-chain networks, brush-like networks are determined by three independent structural parameters: main chain length, side chain length, and grafting density. This transforms the one-dimensional parameter space of synthetic elastomers into a multidimensional landscape of available correlations. However, for the synthesis of multi-arm stars and for obtaining dense molecular brushes, well-organized narrowly dispersed functional oligomers of various structures are needed as building blocks.

Siloxane telechelics with various organic surroundings are the earliest well-defined structure products in this class of macromolecular compounds; they are used as starting reagents for the directed molecular design of siloxane polymers and materials for various applications, the importance of which is growing every day [28].

Linear organosiloxane telechelics have been obtained from the beginning of the development of the chemistry of siloxane polymers in the synthesis of high-molecular products, both by polycondensation of difunctional monomers and by ring-opening polymerization of diorganocyclosiloxanes with ionic initiators. Variation of organic substituents in the structure of polydiorganosiloxane telechelics significantly affects the properties of the obtained products; for example, the introduction of aromatic groups increases the thermal, oxidative, and radiation stability, and the introduction of long alkyl groups leads to an improvement in lubricating properties but reduces their thermal stability. The introduction of highly polar 3,3,3-trifluoropropyl substituents increases the resistance of the polymer to the action of nonpolar solvents while maintaining thermal and thermo-oxidative stability [29].

Currently, a wide range of synthetic approaches are used to obtain organosiloxane telechelics of a given structure, including polycondensation reactions, telomerization, ring-opening polymerization, hydrosilylation, click reactions, etc. The most used, especially on an industrial scale, are the polycondensation and polymerization ROP methods, which are also the most studied processes. The method of hydrolytic polycondensation of difunctional diorganosilanes leads to the formation of a mixture of telechelics and cyclic products, and linear polymers usually have a low molecular mass and a wide molecular mass distribution. For obtaining high-molecular telechelics in this case, the second stage of equilibration and further post-condensation is required [30,31,32,33,34,35,36]. The polycondensation method, on the one hand, is more economical since it uses functional diorganosilanes as the starting compounds and does not require the preparation of starting cyclic structures. However, this method is less suitable for obtaining a well-defined structure, amenable to regulation, and is accompanied by the need for additional stages to obtain high-molecular structures. One of the important exclusions to the general rule is the synthesis of telechelics in an active medium followed by post-condensation using catalysts [37].

An alternative to the polycondensation method for obtaining polydiorganosiloxanes is ring-opening polymerization of diorganosiloxanes (ROP) in the presence of initiators—nucleophilic or electrophilic reagents. In our brief review, we want to consider the current achievements in the field of obtaining polydiorganosiloxane telechelics and monofunctional PDMS, as well as well-organized branching centers by the ROP mechanism and catalytic rearrangement. This is one of the first and most important reactions in the polymer chemistry of silicones, and remains so at the present time.

## 2. Preparation of Diorganosiloxane Telechelics by the ROP Method

**Figure 1:**
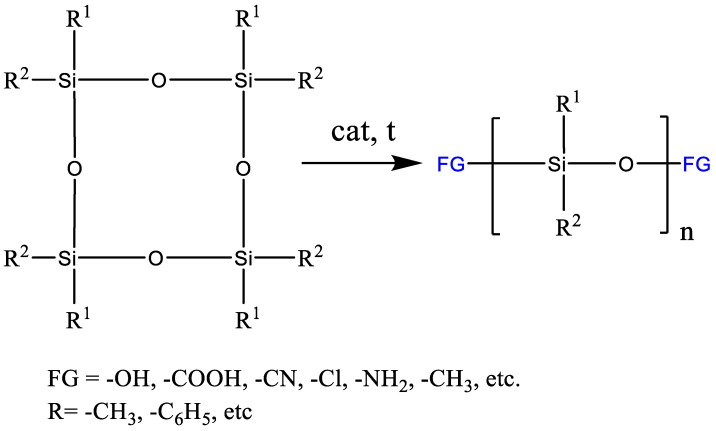
General scheme for the production of polyorganosiloxanes by the ROP mechanism.

The ROP process makes it possible to synthesize polydiorganosiloxanes, including high molecular weight polydiorganosiloxanes, under appropriate conditions with a given molecular weight and a narrow molecular weight distribution (Figure 1). The molecular weight of the polymer is quite effectively controlled by both the amount of introduced initiator and a chain terminator. The usage of a chain terminator containing latent functional groups, for example, vinyl ones, makes it possible to synthesize telechelics with various types of terminal functional groups.

In the middle of the last century, a large number of detailed studies of the regularities and mechanisms of polymerization processes with the opening of cyclosiloxanes were carried out. Next, we briefly consider the most important results.

It is known that the possibility of the process proceeding is determined by the magnitude of the change in the Gibbs free energy ΔG = ΔH − TΔS and is possible only in the case of ΔG < 0. The Si–O bond energies in dimethylcyclosiloxanes with *n* > 3 and in linear dimethylsiloxanes are close to each other [38,39], and the enthalpy change ΔH in the reaction is close to zero. Thus, the driving force of polymerization is mainly the change in entropy, leading to negative values of ΔG. The entropy gain during polymerization in the case of dimethylsiloxanes is due to the higher flexibility of the linear polydimethylsiloxane chain compared to its mobility in cyclosiloxanes, which leads to higher variance in linear polymers [40]. A change in the flexibility of the siloxane chain with a change in the organic surrounding has a significant effect on the course of the process.

With an increase in the volume and polarity of the substituents at the silicon atom, the process leads to a higher yield of cyclosiloxanes. This is due to a relative decrease in the entropy of the polymer because of an increase in the interchain interaction and, accordingly, a decrease in the mobility of polymer chain segments [41]. During the bulk polymerization of [SiR(CH_3_)O]n cyclosiloxanes, the equilibrium concentration of the polymer, depending on the nature of the R substituent, decreases in the row: R = H > CH_3_ > CH_2_CH_3_ > CH_2_CH_2_CH_3_ ≈ C_6_H_5_ >> CH_2_CH_2_CF_3_ [42].

Thus, the polymerization of organocyclosiloxanes by the ROP method behavior depends on the type of organic radicals at silicon atoms, the number of units in the structure of the initial cyclosiloxane, and the nature of the initiator.

### 2.1. Preparation of Organosiloxane Telechelics by Anionic ROP

Anionic ring opening polymerization (AROP) under the action of various nucleophilic reagents is widely used for the synthesis of high molecular weight polydiorganosiloxane telechelics with various organic surroundings of the siloxane chain [28,43].

**Figure 2:**
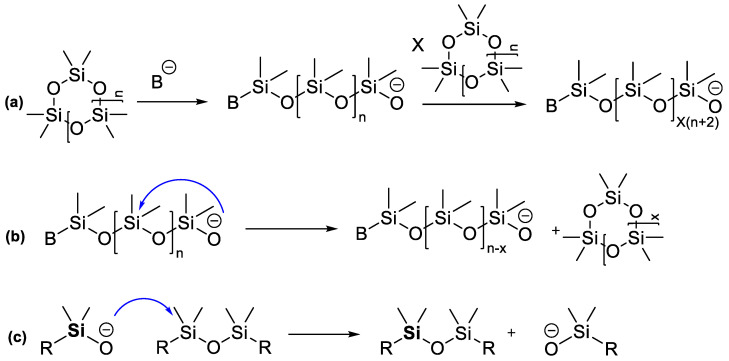
AROP of cyclosiloxanes: (**a**) initiation reaction by formation of silanolate anions and propagation, (**b**) back-biting reaction on a growing PDMS chain; (**c**) chain-transfer reaction.

In the process of cyclosiloxane opening and chain growth (Figure 2a), side processes may occur: depolymerization due to the breaking of the linear chain by the active center (backbiting reaction) (Figure 2b) with the formation of low molecular weight cyclic products, and chain transfer reaction (Figure 2c), in which the terminal active site attacks the siloxane bond of another polymer chain, leading to a redistribution of macromolecules, which is also called equilibration.

#### 2.1.1. AROP Initiators

Hydroxides of alkali metals, quaternary ammonium, and phosphonium bases and their derivatives, siloxanolates are most frequently used as process initiators, which leads to the formation of terminal silanolate anions (Figure 2a) that are active centers in the polymerization reaction.

Potassium hydroxide is one of the first initiators used for ROP of cyclic siloxanes. Its usage dates back to 1948, when ring-opening polymerization with alkali metals was first patented. With its usage, octamethylcyclotetrasiloxane was converted into a high molecular weight (HMW) polymer by heating for two hours at T = 140 °C. The polymer contained 13–15% low molecular weight volatile products; the MW of the main fraction varied from 100,000 at 10% conversion to 1,000,000 at equilibrium [44]. However, the active end groups remaining in the system caused the process of depolymerization at high temperatures; the polymer lost 99% of its mass when kept for 24 h at 250 °C. The formation of a stable product was achieved by neutralizing the product as quickly as possible.

Already in early works, it was demonstrated that the rates of polymerization under the action of hydroxide and siloxanolate of the same metal are the same [45,46]. The activity of hydroxides and siloxanolates of alkali metals during bulk polymerization decreases in the series Cs > Rb > K > Na > Li [47]. Tetramethylammonium and tetrabutylphosphonium siloxanolates are comparable in activity to Cs compounds [48]. The great advantage of these compounds is the possibility of their deactivation and complete decomposition when the polymer is heated, which makes it possible to obtain a neutral thermostable polymer without using the end group blocking step.

**Figure 3:**
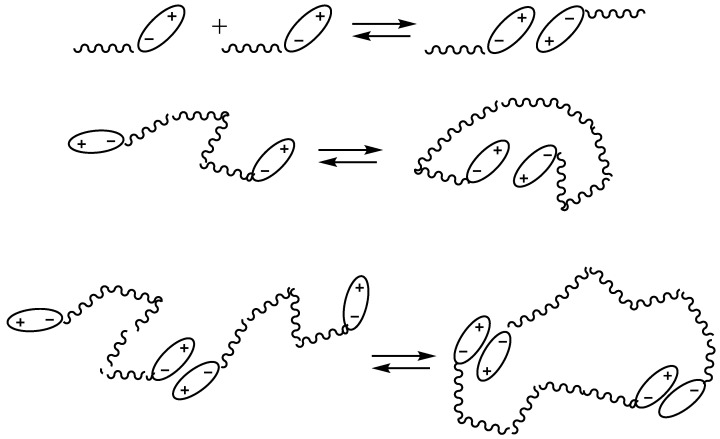
The mechanism of interaction between the anionic center and the siloxane bond.

In the middle of the last century, many research works were devoted to the mechanism of interaction between the anionic center and the siloxane bond. It was shown that silanolates exist in the polymerization system as associates of various sizes. During polymerization under the action of Na, K, and Cs silanolates, apparently, the initiator is present in the system in the active monomeric form and in the form of a low-active binary silanolate complex formed both intermolecularly and within one macromolecule (Figure 3) [49].

The size of associates significantly depends on a specific alkali metal atom and affects both the rate of polymerization and the occurrence of side processes [50]. An increase in the polarity of the polymerization medium by changing the nature of the solvent or introducing small amounts of polar additives leads to the destruction of inactive metal associates and the formation of solvated ion pairs, which significantly increases the rate of polymerization of cyclosiloxanes [51,52,53]. Butyllithium is the most popular catalyst for non-equilibrium anionic ROP [53,54]. Lithium compounds used as initiators, such as n-, sec- and tert-butyllithium, have a number of features that significantly differ their behavior in ring-opening anionic polymerization reactions, especially in the case of D3. In a non-polar medium, when D3 and BuLi react, the reaction products are exclusively the corresponding BuDLi and the remaining cyclosiloxane [55]:

BuLi + [Me_2_SiO]_3_ → BuSiMe_2_OLi + 2/3[Me_2_SiO]_3_

Apparently, due to the abnormally low activity of the lithium counterion and the high tendency to aggregation, the formation of inactive associates leads to the appearance of the first silanolate groups. Then the attack proceeds only along the activated siloxane bond adjacent to the silanolate bond until they are completely exhausted, and the process of opening no cycles. This feature formed the basis for the creation of a two-stage procedure for carrying out ROP D3 polymerization. The first stage is carried out in solution in a non-polar medium to convert active groups into a silanolate form with equal activity, and at the second stage, with the introduction of activating polar agents, the process continues until the initial cyclosiloxanes are exhausted. This approach makes it possible to obtain monomodal narrowly dispersed polydimethylsiloxane.

**Figure 4:**
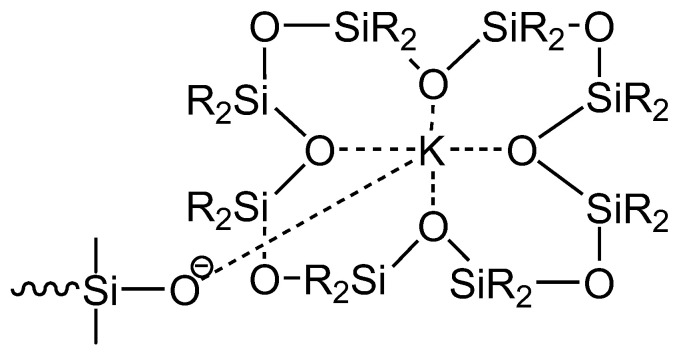
Metal coordination by oxygen atoms of the cyclosiloxane molecule.

An interesting effect of solvation and separation of associates of active sites, leading to an increase in the polymerization rate, is observed in the polymerization of dimethylcyclosiloxanes with *n* > 5. The rate of polymerization of cyclosiloxanes with *n* = 7 and 8 is more than 100 times higher than the rate of polymerization of octamethylcyclotetrasiloxane. This acceleration is explained by the coordination of the metal by the oxygen atoms in the cyclosiloxane molecule, similarly to the interaction between the cation and the crown ether (Figure 4) [56]. However, this effect was not subsequently reproduced or somehow confirmed. Apparently, the “crown” is instantly exhausted during the reaction process.

A polymer with a polydispersity index close to 1 was obtained using strong complexing agents as polar additives, for example, crown ethers and cryptates of the appropriate size [53]. The complexes were formed in these cases with the lithium counterion of the initiator. It led to the complete destruction of their aggregates and a significant increase in the polymerization rate in the almost complete absence of depolymerization processes. This technique was also successfully used in the polymerization of hexaethylcyclotrisiloxane, which is much less active than hexamethylcyclotrisiloxane [52].

The results of the study of AROP with a lithium counterion in the presence of traces of water are of practical importance. In this case, even when using a monofunctional, widely used organolithium initiator, the formation of a telechelic occurs due to the fast exchange reaction ~SiMe_2_-O-Li + HOH ↔ ~SiMe_2_-OH + Li-OH and subsequent opening of cyclosiloxane in situ with lithium hydroxide, with the formation of a macromolecule with two terminal functional groups. It was shown that between the active end groups ~SiMe_2_-OLi and ~SiMe_2_-OH there is a rapid exchange and the formation of an associate, and these ends are equally active in the process. The ratio of the amounts of water and lithium initiator in the system determines the rate of the process; an increase in the amount of water leads to an increase in the induction period. In this case, when using a monofunctional initiator, a monomodal telechelic is formed at [init] << [HOH]. In the case of [init]~[HOH], the polymerization product is bimodal with the presence of monofunctional macromolecules [57].

The most important class of initiators are tetraalkylammonium and tetraalkylphosphonium hydroxides and silanolates, which are used both in earlier publications and today [58,59,60]. The advantage of this series of catalysts is that, compared to other catalysts, they are quite easily removed from the polymer—they undergo thermal decomposition with the formation of volatile by-products and a neutral, thermally stable polymer.

Another group of catalysts is quaternary phosphazene bases. They catalyze the polymerization of D4 in exactly the same way and decompose at high temperatures in the same way. Decomposition products are non-toxic and do not react with the polymer [61,62,63].

**Figure 5:**
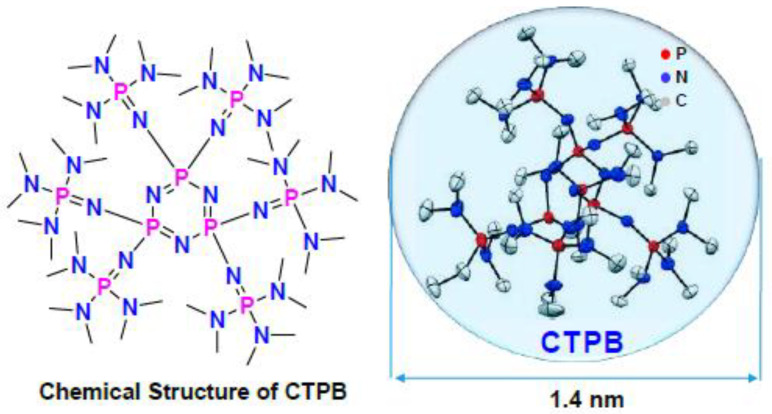
Structure of a trimeric phosphazene base (CTPB) (adapted with permission from Ref. [64]. 2019, Shi, J).

Modern studies also mention new types of AROP catalysts. Jinfeng Shi et al. showed that the organic cyclic trimeric phosphazene base (CTPB) (Figure 5) is highly efficient for the ring-opening polymerization (ROP) of octamethylcyclotetrasiloxane (D4) and the copolymerization of D4 with octaphenylcyclotetrasiloxane (P4) under mild conditions. The polymerization proceeds rapidly, and the obtained polymers have a rather high molecular weight (Mn up to 1,353,000 g/mol). For the copolymerization of D4 and P4, it is easy to prepare copolysiloxanes with different contents of diphenylsiloxane (up to 64 mol%). There was no observed Q- or T-branching for copolysiloxanes in all cases according to 29Si NMR analysis, indicating good ROP control with the current CTPB/BnOH catalyst system. DSC analysis confirms that the copolysiloxanes are amorphous and Tg increases with increasing diphenylsiloxane content in the polymer chain. TGA analysis shows that the increase in thermal stability is achieved by introducing a diphenylsiloxane unit [64].

**Figure 6:**
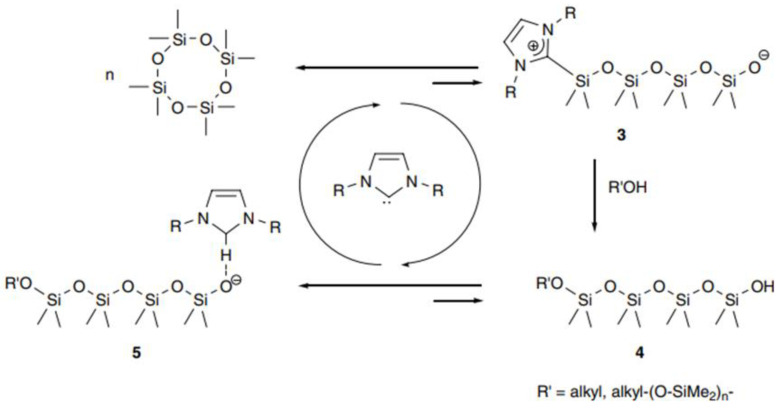
Proposed mechanism of ROP of D_4_ (adapted with permission from Ref. [65]. 2007, Rodriguez, M).

Recently there have been reports on the use of N-heterocyclic carbenes or bicyclic guanidines as AROP initiators. For example, Marta Rodriguez et al. report that N-heterocyclic carbenes are effective ROP catalysts for cyclotetrasiloxane D4 under mild conditions. Interestingly, a system using primary alcohols (MeOH and BnOH) more effectively controls the molecular weight of the polymer; the molecular weight of silicone polymers can be controlled by simply changing the amount of alcohol initiator. Due to neutral conditions, the only by-products are a catalytic amount of moisture sensitive NHC and volatile alcohol, which are easily removed (Figure 6) [65].

However, other types of catalysts are also mentioned in the current literature. For example, Bashim Yactine used potassium vinyldimethylsilanolate (KVDMS) and potassium trimethylsilanolate (KTMS) catalysts in his work to perform ROP. Anionic polymerization of D3 initiated by potassium vinylsilanolate proved to be very efficient for the synthesis of difunctional and monofunctional polydimethylsiloxanes, respectively [66].

**Figure 7:**
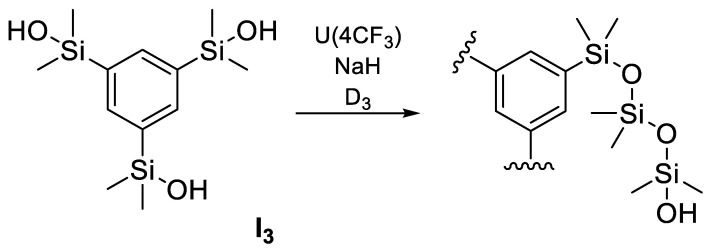
ROP of D_3_ initiated from I_3_ with U(4CF_3_).

Oka et al. used the urea anion as a catalyst for ring-opening polymerization (ROP) of a cyclic siloxane initiated from silanols, which allowed control of the molecular weight and fineness of the final product. ROP of D3 was initiated with a trifunctional silanol (I_3_) (Figure 7), to form star-shaped polysiloxanes. Trifunctional silanol I_3_ is relatively poorly soluble in THF; deprotonation of I_3_ with NaH in THF led to the formation of a precipitate. However, the addition of U(4CF_3_) to this mixture solubilized the ionic species to give a homogeneous compound. The D3 conversion reached 96% in 60 min, although the dispersity increased slightly. Urea anion catalysts are particularly useful for producing lower molecular weight polysiloxane stars. Moreover, the combination of silanols and urea anion catalysts overcomes the solubility problem arising from the polarity mismatch between the initiators, D3 and PDMS [67]. However, this process remains rather complicated.

**Figure 8:**
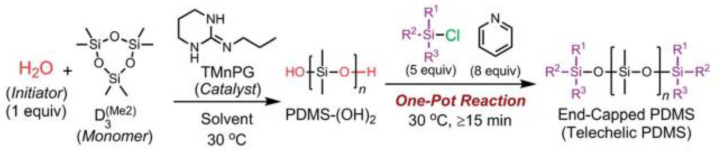
Scheme for obtaining PDMS-telechelics by the AROP mechanism initiated by water and TMnPG (reprinted with permission from Ref. [68]. 2018, Fuchise, K).

An organocatalytic controlled or living ROP of cyclotrisiloxanes using water as an initiator, strong organic bases as catalysts, and chlorosilanes as blocking agents was developed by the Keita Fuchise group for a convenient and efficient method for the synthesis of linear polysiloxane telechelics and their copolymers (Figure 8). It was shown that guanidines B, namely guanidines containing the R–N=C(N)–NH–R’ unit, showed the highest catalytic activity depending on their Brønsted basicity among the tested organic bases. In particular, TMnPG, monocyclic guanidine B, was the best in terms of its high catalytic activity and low number of side reactions [68].

The polymerization rate of AROP depends on the nature of the initiator, the polymerization medium, and the selected monomer. However, the key factor controlling the kinetics of ring-opening polymerization is the counterionic interaction of silanolates leading to the formation of aggregates that are inactive in AROP [45].

The polymerization itself can be carried out in the bulk, in solvents, or in emulsion. However, the solubility of the initiator in the reaction medium plays an important role in the reaction kinetics. Suitable solvents are liquid hydrocarbons. In some examples, THF is used as a solvent in combination with a solid counterion [69].

#### 2.1.2. Influence of the Structure of the Initial Organocyclosiloxane on the AROP Process

The next parameter that determines the course of the AROP process is the structure of the starting organocyclosiloxane and the nature of the organic substituents at the silicon atom.

The influence of the size of the initial cyclosiloxane is clearly manifested in the case of the most widely used dimethylsiloxane cyclosiloxanes, hexamethylcyclotetrasiloxane (D3) and octamethylcyclotetrasiloxane (D4). The difference between these compounds and, accordingly, the conditions and results of their polymerization processes is quite large.

The similarity of the binding energies in dimethylcyclosiloxanes with *n* > 3, primarily in octamethylcyclotetrasiloxane and in linear dimethylsiloxanes, leads to the fact that the process is ongoing exclusively due to the thermodynamic component. Ionic active centers attack and break the Si–O bonds both in dimethylcyclosiloxane and in the resulting linear polymer, carrying out the depolymerization process parallel to polymerization. The result is a mixture of linear and cyclic siloxanes with different ring sizes. In the system, over time, an equilibrium is established between the polymer and the mixture of cyclosiloxanes. The equilibrium position in this case does not depend on temperature, because ΔH~0, same as the nature of catalyst and solvent [70].

The process of polymerization of hexamethylcyclotrisiloxane, which has a significant intensity of the three-link cycle, looks different, as a result of which the thermal effect of polymerization is ΔH~3–4 kk/mol. The presence of an energy gain during the opening of hexamethylcyclotrisiloxane makes it possible, under certain conditions, to carry out polymerization in a nonequilibrium mode, which excludes depolymerization reactions and interchain exchange. The rate of polymerization of hexamethylcyclotrisiloxane under comparable conditions is almost 100 times higher than the rate of polymerization of octamethylcyclotrisiloxane. This method for the synthesis of polydimethylsiloxane makes it possible to obtain polymers with a controlled molecular weight, specified end groups, and a narrow molecular weight distribution (Mw/Mn = 1.0–1.2) [53,71,72], and the resulting dimethylsiloxane telechelics are widely used to obtain siloxane block copolymers polymer networks. The use of appropriate blocking reagents makes it possible to obtain polymer products with specified end groups [72]. The disadvantage of this method for obtaining polydimethylsiloxane telechelics is the need to obtain hexamethylcyclotrisiloxane, which is a more expensive monomer than unstrained cyclosiloxane, and stringent requirements for the process conditions for the purity of the system and temperature. Thus, from a commercial point of view, the polymerization of octamethylcyclotetrasiloxane is preferred [73,74].

The process of polymerization of diphenylcyclosiloxanes, both 6- and 8-membered, is completely different from other. Polydiphenylsiloxanes (PDFS) have very high thermal and radiation resistance, a very high melting point (about 265 °C), and mesophase properties [75], in connection with which they are of great interest; however, the preparation of telechelics and, in general, polymers of this nature remained an unsolved problem for a very long time. The extremely high tendency to cyclization of the diphenyl chain, caused by the steric factor, apparently playing a decisive role in this case. In all polycondensation and polymerization processes, the reaction product was either tetraphenyldisiloxanediol, or further octaphenylcyclotetrasiloxane [76]. Under anionic conditions, the polymerization and depolymerization processes proceed simultaneously and at the same rate and lead to the formation of low molecular weight cyclic products. Moreover, only in this case, the differences in the tension of the initial 6- and 8-membered diphenylcyclosiloxanes do not play a significant role: apparently, the thermodynamic unfavourability of the polymer chain leads to its absence in the products of the equilibrium process. The implementation of a nonequilibrium process without a significant occurrence of depolymerization was only possible under the conditions of solid-state polymerization of hexaphenylcyclotetrasiloxane at temperatures close to the melting point of the cycle, but not reaching it. It was shown that polymerization proceeds under heterogeneous conditions; the reaction proceeds inward from the surface of HPTS crystals and leads to the formation of a crystalline polymer, with polymerization and crystallization proceeding sequentially [77]. In this case, the crystallinity of polymerized PDPS samples is inversely proportional to its specific viscosity [78].

**Figure 9:**
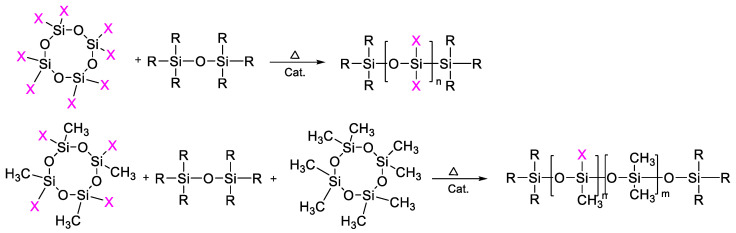
Obtaining functional PDMS by the ROP mechanism.

From the point of view of the arrangement of functional groups, the literature considers the preparation of two types of siloxane telechelics obtained by ROP: siloxane oligomers with functional groups directly bonded to terminal silicon atoms (Si–X) and siloxane oligomers with an organofunctional end (Si–R–X). Moreover, in addition to functional ends, functional groups can also be attached to the backbone of the polysiloxane (Figure 9). This can be achieved by the aforementioned ROP of cyclotetrasiloxane as well as by catalytic rearrangement reactions. However, instead of the usual D4 or D3 that give known PDMS, the methyl group(s) attached to the Si atom of cyclic siloxanes can be replaced with different functional groups before polymerization [79,80].

The above processes proceed according to one of three different basic reaction mechanisms: cationic, anionic, or coordination intercalation [81].

The next factor that has a significant effect on the course of polymerization is the nature of the organic groups at the silicon atoms of siloxane cyclosiloxanes. The introduction of electron-donating substituents—longer hydrocarbons than the methyl group—reduces the rate of anionic polymerization of cyclosiloxane, while electron-withdrawing substituents—alkenyl, aromatic, phenyl, 3,3,3-trifluoropropyl or cyanoalkyl groups—increase the rate of polymerization [82,83,84]. In this case, electron-withdrawing substituents at the silicon atom during anionic polymerization lead to the formation of a siloxanolate anion with a lower nucleophilic activity, which somewhat levels out the increase in the activity of cycles during polymerization. In addition, the steric influence of bulky radicals leads to low reactivity of the corresponding cyclosiloxanes [85].

#### 2.1.3. Preparation of Monochelical PDMS by AROP

**Figure 10:**
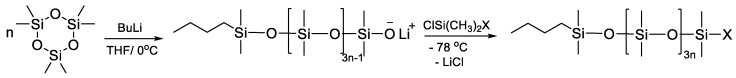
Scheme for the preparation of polydimethylsiloxane monochelics by the AROP mechanism.

Monofunctional PDMS, also referred to as “macromonomers”, are usually synthesized by living anionic polymerization of hexamethylcyclotrisiloxane. These experiments were first carried out in the 1960s by Bostick [86] and Lee [53], and showed the possibility of obtaining monofunctional polydimethylsiloxanes with controlled molecular weight and narrow molecular weight distribution by anionic polymerization of hexamethylcyclotrisiloxane (D_3_) using lithium silanolate salts (R-Li^+^) or lithium as initiators in the presence of promoters such as THF or diglyme (Figure 10).

Theoretically, anionic polymerization can be carried out using D4, however, the reaction will tend to equilibrate even at low conversions. Accordingly, the resulting polymers have a relatively broad molecular weight distribution and contain appreciable amounts of macrocyclic oligomers [53]. By using a cyclic trimer as a starting material, siloxane redistribution processes other than the desired ring-opening chain propagation reaction can be almost completely eliminated. This is mainly due to the ring tension in the D3 monomer, which significantly increases its reactivity towards anionic initiators. Selectivity is further enhanced by the use of initiators in which lithium is the counterion. Initiators containing lithium counterions are preferable to counterions of other alkali metals because of the lower catalytic activity of lithium in siloxane redistribution reactions [87]. Monofunctional oligomers are characterized by low molecular weight (500–20,000 g*mol^−1^).

Using the ROP D_3_ mechanism in the presence of BuLi, hydridesilyl monofunctional PDMS [88,89], acrylate monofunctional PDMS [90], and amine-containing monofunctional PDMS [91] are obtained.

Vysochinskaya Y.S. et al. [92] synthesized monofunctional vinyl PDMS from D_3_ in the presence of BuLi as a catalyst and dimethylvinylchlorosilane as a blocking agent. The resulting compounds were used to prepare star-shaped polymers by hydrosilylation reaction with cyclic nuclei with Si-H groups in the presence of Karstedt’s catalyst.

**Figure 11:**
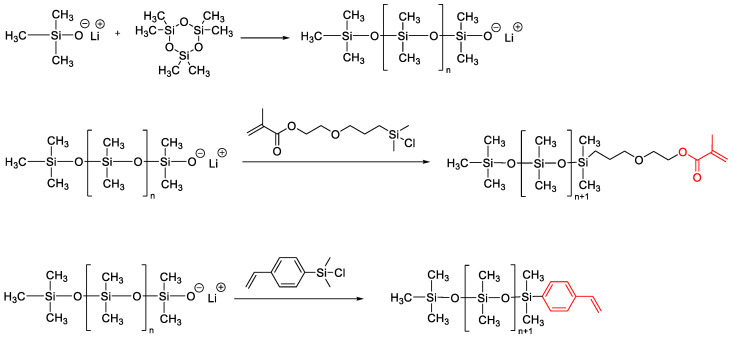
Obtaining monofunctional macromonomers of the styrene and methacrylate type by the AROP mechanism.

**Figure 12:**
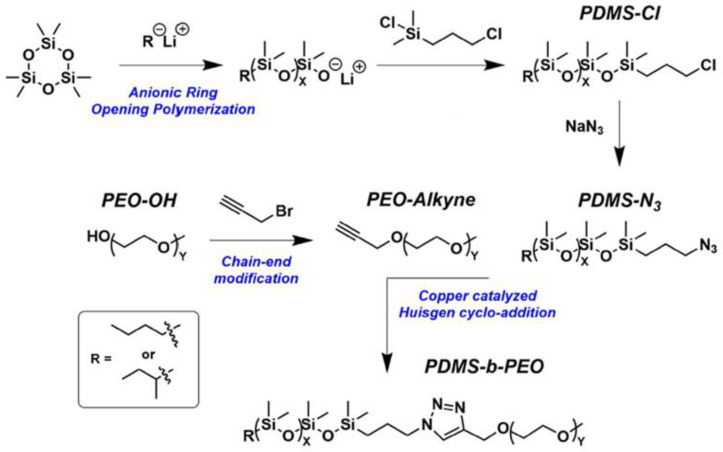
General synthesis procedure to obtain PDMS-b-PEO copolymers (adapted with permission from Ref. [94]. 2019, Fauquignon, M).

Kawakami Y. et al. [93] used the same method to obtain monofunctional macromonomers of the styrene and methacrylate type, which are the starting compounds for obtaining graft polymers (Figure 11):

Martin Fauquignon et al. synthesized a series of PDMS-b-PEO diblock copolymers of various molar masses and hydrophilic mass fractions. He used monofunctional 3-chloropropyl-PDMS which were synthesized by anionic ring-opening polymerization of the hexamethylcyclotrisiloxane (D3) monomer in anhydrous THF at 80 °C (Figure 12). Butyllithium was used as the initiator, functionalization of the end of the chain was obtained using chloro-(3-chloropropyl)dimethylsilane as a termination agent [94]. The subsequent conversion of the chloropropyl group to the azidopropyl group made it possible to obtain poly(dimethylsiloxane)-block-poly(ethylene oxide) (PDMS-b-PEO) diblock copolymers by click chemistry. These polymers are applicable in the developing field of hybrid polymer/lipid vesicles. According to the same scheme, monofunctional azide PDMS were obtained in [95,96] for the subsequent preparation of copolymers.

**Figure 13:**
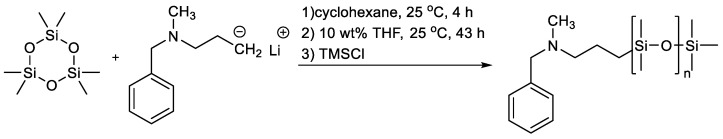
Anionic Polymerization of D_3_ Using 3-[(N-Benzyl-N-methyl)amino]-1-propyllithium.

**Figure 14:**
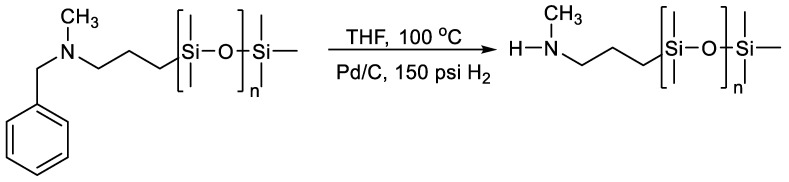
Synthetic Methodology for the Deprotection of the Amine Functionality on PDMS.

The functional end group can be introduced either by the organo segment of the initiator or by the chlorosilane molecule. Functionalized initiation has been reviewed by Casey L. [97]. A number of poly(dimethylsiloxane) homopolymers in the molar mass range from 2400 to 15,000 have been synthesized using 3-[(N-benzyl-N-methyl)amino]-1-propyllithium (Figure 13). The protecting group on PDMS was quantitatively removed by hydrogenolysis to give a secondary amine (Figure 14):

A recent review by Goff J., Sulaiman S., and Arkles B. [98] is dedicated to the production of monofunctional PDMS and their application. However, the use of monofunctional PDMS in copolymerization reactions is rather limited. It is much more common to obtain difunctional terminal (telechelic) silicone oligomers, which are the starting compounds for a wide range of silicone copolymers.

#### 2.1.4. Obtaining Functional Telechelics by AROP

For the further usage of organosiloxane telechelics, the nature of the terminal functional groups is an important parameter. Since the end of the last century, the most important PDMS-telechelics have been obtained by the AROP mechanism: vinyl-functional [99,100] and amine-functional [101,102,103], etc.

**Figure 15:**
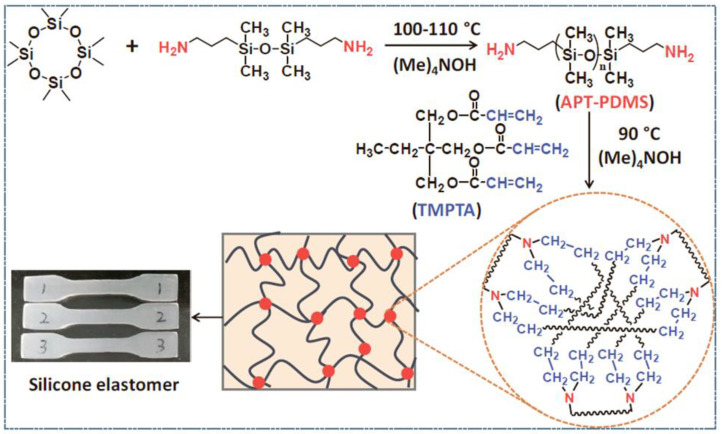
Illustration of the synthetic strategy and structure of silicone elastomer (adapted with permission from Ref. [104]. 2018, Li, X).

Obtaining new functional PDMS-telechelics is also relevant in our time. Li X. et al. [104] obtained aminopropyl terminated polydimethylsiloxane in the presence of a tetramethylammonium hydroxide catalyst by the AROP mechanism to subsequently obtain silicone elastomers with good mechanical properties, as well as high self-healing efficiency through a simple amino-ene addition reaction according to Michael (Figure 15). A basic TMAH catalyst is added to the system to provide a dynamically crosslinked silicone elastomer network. Tensile tests show that the silicone elastomer has very good mechanical properties for an unfilled silicone composition with a tensile strength and an elongation at break of 1.08 ± 0.06 MPa and 206.10 ± 9.55%, respectively. After holding at 105 °C for 24 h, the tensile strength of “broken” specimens can recover 91% of their original strength, and specimens cut into multiple pieces can regain their original shape.

Also, the preparation of aminopropyl terminated polydimethylsiloxane by the anionic ROP mechanism is reported in the work of V.V. Gorodov [105]. The resulting oligomeric amine-containing PDMS was subsequently treated with itaconic acid in an o-xylene solution in the presence of anhydrous magnesium sulfate as a dehydrating agent. Thus, telechelic oligodimethylsiloxanes with 4-carboxypyrrolidone fragments were synthesized and their thermal and rheological properties were studied. These polymers have been found to be prone to the formation of smectic-type mesophases. The introduction of carboxypyrrolidone groups into the siloxane chain significantly increases the viscosity and activation energy of the viscous flow of oligodimethylsiloxanes.

**Figure 16:**
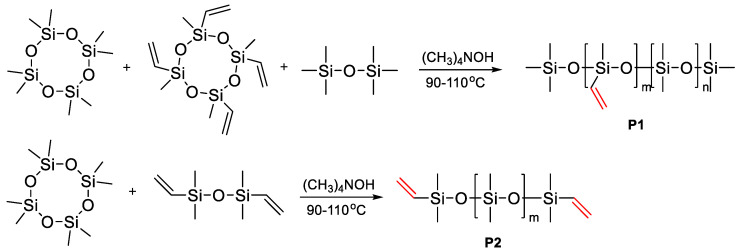
Synthesis of the functionalized polysiloxanes (P1, P2).

The group of Zuo Y. [106] obtained vinyl functional PDMS telechelics, as well as vinyl functional copolymers (Figure 16) by the AROP D4 mechanism in the presence of a tetramethylammonium hydroxide catalyst. Then these polymers were functionalized with N-acetyl-L-cysteine using thiol-ene chemistry, subsequently forming new transparent, luminescent silicone elastomers. Luminescence centers were formed by complexation of lanthanide ions into a functionalized polysiloxane.

**Figure 17:**
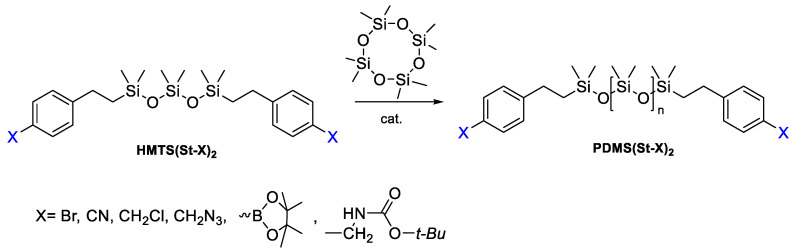
Common scheme of the polymerization of octamethylcyclotetrasiloxane with functional trisiloxanes as stoppers.

**Figure 18:**
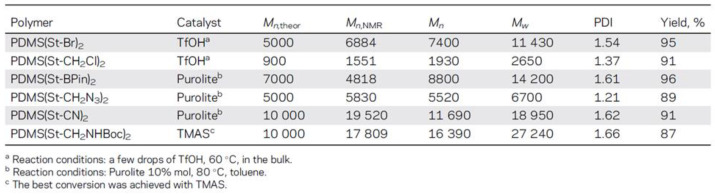
Reaction Conditions and Molecular-Weight Characteristics of Synthesized Telechelic Bifunctional PDMS (adapted with permission from Ref. [107]. 2018, Drozdov, F.).

It should be noted that in modern studies on the ROP mechanism, it is possible to obtain telechelics with new functional groups. For example, F.V. Drozdov et al. reported the production of a number of unusual functional PDMS telechelics by the ring-opening polymerization D4 mechanism, using various functional trisiloxanes as a stopper and TfOH/Purolite/tetramethylammonium silanolate (TMAS) as a catalyst (Figure 17). If acid-resistant functional groups were present in the monomer, TfOH or Purolite was used as an acid catalyst and bulk polymerization was carried out. Otherwise, a basic catalyst was used and the reaction was carried out in toluene. For the polymerization of PDMS(St-CH_2_NHBoc)_2_, a number of catalysts were used: TfOH, Purolite, tetramethylammonium silanolate (TMAS), and KOH. In the case of acidic catalysts (TfOH and Purolite), only an insignificant part of the starting monomer participated in the polymerization even for 48 h at 60 °C. Using TMAS, a polymer with high conversion and molecular weight distribution was obtained. Thus, PDMS were obtained in the range of molecular weights from 1500 to 30,000 (Figure 18). The synthesis of not only symmetrical oligosiloxanes but also asymmetric hydridosiloxane-containing analogs has potential applications as precursors for the preparation of functional siloxane derivatives by the hydrosilylation reaction. Thus, symmetrical or asymmetric telechelic oligo- or polydimethylsiloxanes with different functional groups are suitable as AA-type blocks for further synthesis of block copolymers [107].

**Figure 19:**
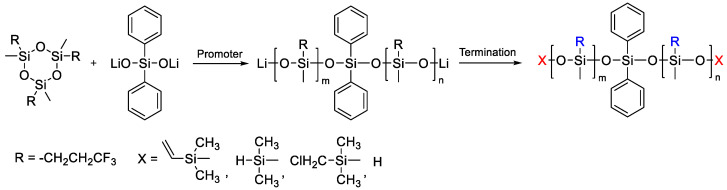
Synthetic routes of PMTFPS with different end groups.

Moving on nonequilibrium processes, it is worth noting the work of Fei H.F. et al. [108], where they studied the anionic ring-opening polymerization of 1,3,5-tris(trifluoropropylmethyl)cyclotrisiloxane in bulk using dilithium diphenylsilanediolate as initiator (I); and N,N-dimethylformamide (DMF), bis(2-methoxyethyl)ether (Diglyme), and 1,2-dimethoxyethane (DME) as promoters (P) (Figure 19). A detailed study of the polymerization kinetics with various molar ratios of promoter to initiator ((P)/(I)), which were equal to 2.0, 4.0 and 6.0, showed that the yield of linear polymers was highest when (P)/(I) 2.0 for all promoters, among which DME was the most effective in suppressing side reactions. The reaction initiated by DME had a very wide “cutoff window” with the highest linear polymer yield and a very narrow molar mass distribution. PMTFPS with end groups such as vinyl, hydroxyl, hydrogen and chloromethyl were obtained and characterized by ^1^H NMR, ^29^Si NMR, and FT-IR. Vinyl-terminated polymers showed higher thermal stability than hydroxyl-terminated polymers under nitrogen atmosphere.

In a review by Köhler T. [1], the process of obtaining OH-terminated polydimethylsiloxanes by the ROP mechanism is considered in detail. US Pat. No. 5475077 describes a batch process for the synthesis of certain OH-terminated silicones by AROP. To do this, a mixture of cyclic siloxanes (mainly D_3_, D_4_, and D_5_) is subjected to interaction with an aqueous solution of KOH at 170 °C. The reaction mixture is purged with steam to remove air and other gases. After keeping the reaction mixture for some time at this temperature and under a certain pressure of water vapor, ethylene chlorohydrin is added to neutralize KOH, which precipitates in the mixture in the form of a salt. The reaction product is then desorbed and silanol-terminated PDMS is obtained. The viscosity of the resulting polymer, which depends on the molecular weight and polydispersity, is controlled by the water vapor pressure in the vessel. The higher the water vapor pressure, the lower the viscosity of the resulting product.

**Figure 20:**
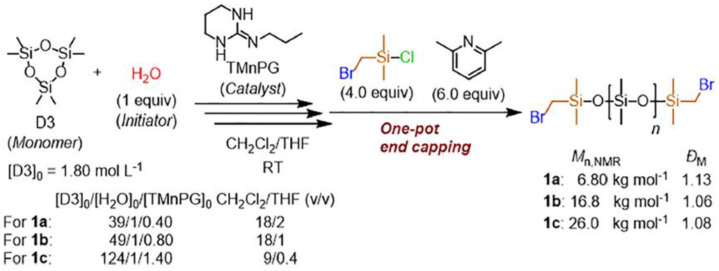
Synthesis of α,ω-chain-end-functionalized PDMS with bromomethyl groups (Br-PDMS-Br: 1a/1b/1c) by the controlled ring-opening polymerization of hexamethylcyclotrisiloxane (D3) catalyzed by 1,3-trimethylene-2-n-propylguanidine (TMnPG) (adapted with permission from Ref. [109], 2021, Sato, K.I).

In a recent study by Sato K. et al. [109], the authors obtained of α,ω-chain-end-functionalized PDMS with bromomethyl groups also by the ROP mechanism from D3 in the presence of water and a catalyst from the guanidine series using bromomethyldimethylchlorosilane as a blocking agent (Figure 20). The advantages of this approach include the narrow dispersity of the obtained compounds, a fairly wide range of molecular weights, and the disadvantages are a rather laborious synthesis scheme. The resulting compounds were subsequently converted into azide PDMS for subsequent azide-alkyne cycloaddition reactions, model variants of which are also presented in the work.

**Figure 21:**
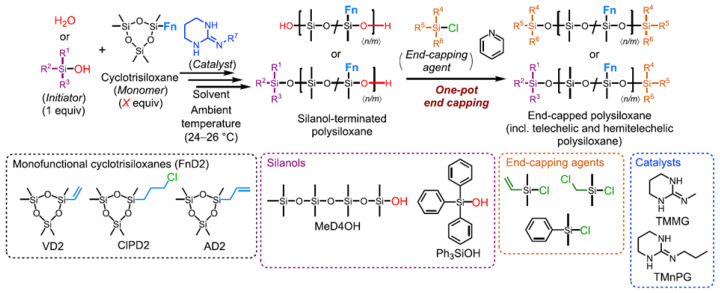
Ring-Opening Polymerization (ROP) of Monofunctional Cyclotrisiloxanes Using Water or Silanols as Initiators and Guanidines as Catalysts (adapted with permission from Ref. [110]. 2021, Fuchise, K.).

In another work by the same group of Japanese scientists [110], functional linear polysiloxanes were obtained, namely vinyl, 3-chloropropyl, and allyl (Figure 21) by an organocatalytic controlled/living ring-opening polymerization (ROP) of monofunctional cyclotrisiloxanes using water or silanols as initiators, guanidines as catalysts, and chlorosilanes as blocking groups. The AROP method has made it possible to obtain polymers with a controlled number average molar mass (Mn), a narrow molar mass distribution, desirable end structures, and a good distribution of side chain functional groups. It can be expected that this convenient new method for the synthesis of linear polysiloxanes with well-defined functionalized side chains will allow the preparation of organosilicon compounds, hybrid materials with a variety of polymer structures, such as block copolymers, star polymers, comb polymers, and surface modified materials, which will ultimately contribute to the development of new advanced materials with improved properties. The vinyl, 3-chloropropyl, and allyl groups on the side chains can be further converted to other structures through a variety of reactions, including hydrosilylation, thiol-ene, oxidation, and nucleophilic substitution.

**Figure 22:**
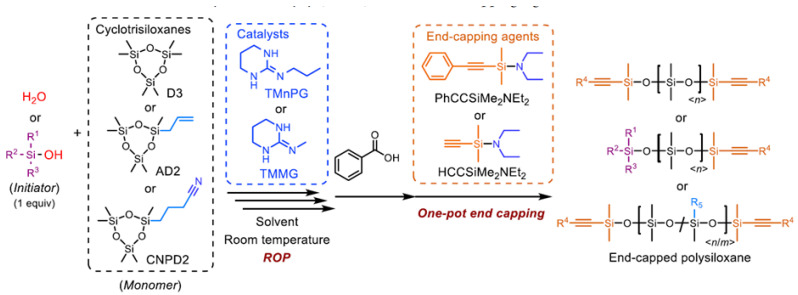
Precise Synthesis of Alkynylsilyl-Terminated Polysiloxanes by ROP of Cyclotrisiloxanes Using Water or a Silanol as an Initiator, Guanidines as Catalysts, and alkynyl(amino)silanes as End-Capping Agents (adapted with permission from Ref. [111]. 2021, Fuchise, K).

The same group of scientists carried out the synthesis of linear polysiloxanes functionalized with disubstituted and monosubstituted alkynyl groups, also by the method of controlled/living ring-opening polymerization of cyclotrisiloxanes using water or silanols as initiators, guanidines as catalysts, and alkynyl (amino) silanes as end blockers agents (Figure 22) [111]. Two alkynyl(amino)silanes, (diethylamino)dimethyl(phenylethynyl)silane and (diethylamino)ethynyldimethylsilane were synthesized by alkynylation of chloro(diethylamino)dimethylsilane. In addition, the resulting alkynyl-terminated polysiloxanes were subjected to non-catalytic and catalytic Huisgen reactions with organoazide compounds. The resulting polysiloxanes can be used in other reactions involving alkynyl groups, especially alkynylsilyl groups. These new polysiloxanes will provide great opportunities in molecular design and for obtaining polymer structures and cross-linked materials, hybrid materials with controlled molecular or network structure, which, in turn, should lead to the development of new advanced materials with improved properties and/or unprecedented functionalities.

Thus, anionic ROP has been a universal method for the preparation of a wide range of functional PDMS telechelics for many years. At present, the reaction remains relevant, and scientists are able to introduce new types of functional groups (for example, azide and acetylene) into the structure of PDMS, which, of course, expands the scope of such compounds.

### 2.2. Preparation of Organosiloxane Telechelics by Cationic ROP

Cationic ROP is also of interest for the preparation of functional PDMS. The advantage of this process is that it can be carried out at a relatively low temperature, the catalyst can be easily deactivated, and the process can also be used to synthesize polysiloxanes having base-sensitive substituents such as Si–H or Si–(CH_2_)_3_–SH [112].

#### 2.2.1. CROP Initiators

The first high molecular weight siloxane polymer was obtained by ring-opening D4 cationic polymerization in the presence of sulfuric acid. Polymerization in the presence of sulfuric acid proceeds in several stages. Acid is usually introduced in an amount of 1–3% (wt.). Polymerization lasts from two to eight hours at room temperature and leads to the formation of low molecular weight polymers, therefore, at the end of polymerization, a small amount of water is added to the system for subsequent growth of molecular weight. However, the polymerization mechanism is complex and is still a subject of debate in the literature due to the fact that some unusual kinetic patterns have been observed. There is a negative order in monomer concentration and a negative activation energy [112,113]. The role of water in the polymerization process is also a matter of debate as it can act as a promoter and inhibitor in CROP [114].

**Figure 23:**
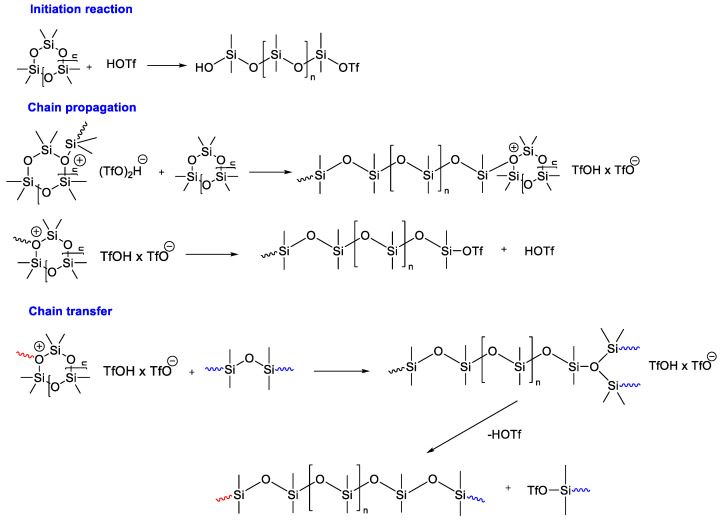
Reaction steps discussed for trifluoromethanesulfonic acid catalyzed polymerization of cyclosiloxanes.

The mechanism of polymerization using trifluoromethanesulfonic acid as an initiator has been studied in more depth [115,116]. It is generally accepted that the Si–O bond is cleaved by strong protonic acids during the initiation of the reaction (Figure 23). Thus, the corresponding silanol based on the silyl ester is formed, which starts chain growth.

Other catalyst systems have been reported in the literature such as HClO_4_, aryl and alkyl sulfonic acids, heterogeneous catalysts such as ion exchange resins, acid treated graphite and acid treated clays, and some Lewis acids such as SnCl_4_ [38,117,118,119,120]. Polymerization in the presence of Lewis acids is a matter of controversy. Strong protonic acids such as HSnCl_5_, the reaction product of a Lewis acid with water or other protonic impurities, are also suggested as catalysts [121]. However, it was reported that some non-protic systems, such as ethyl boron sesquitriflate [122] and antimony chloride vapors-acid chloride pairs [123], are capable of initiating the polymerization of cyclotrisiloxane. However, they have not received wide distribution due to either insufficiently good process control or their high cost.

Other unusual types of catalysts are also mentioned in the literature. V.M. Djinovic at al. synthesized a series of α,ω-dicarboxypropyloligodimethylsiloxanes with a given molecular weight from octamethylcyclotetrasiloxane and 1,3-bis-(3-carboxypropyl)tetramethyldisiloxane (BCPTMDS) using a macroporous cation exchange resin as an acid catalyst. At the same time, the expected molecular weights in the range from 600 to 3500 were achieved with acceptable accuracy. However, the authors did not provide data confirming the effectiveness of this catalyst at higher molecular weights [124].

In Yactine B. [66], acid-treated bentonite (sold under the trade name TONSIL1) was chosen as the CROP catalyst because of its ability to catalyze the polymerization of cyclosiloxanes at a relatively low temperature (typically 70 °C) and because of its easy filtration departments. The novelty here is to compare conventional ROP D4 (and sometimes D^H^4) using a conventional terminating agent with redistribution reactions starting with telechelic PDMS and D4 commonly practiced in the industry. Such methods will make it possible to obtain Si–H or Si–vinyl terminated telechelic homopolymers and copolymers [125,126].

**Figure 24:**
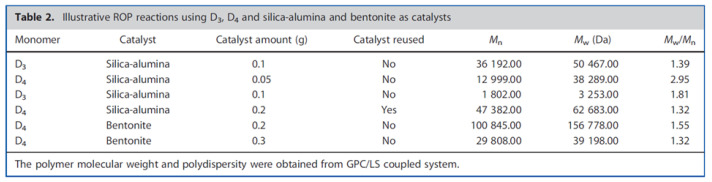
Chemical and textural properties of silica-alumina and bentonite before and after acid treatment (adapted with permission from Ref. [127]. 2012, Vallejo-Montesinos, J.) Center.

Javier Vallejo-Montesinos and colleagues have used synthetic and natural silicon aluminates as inorganic acid catalysts for ring-opening polymerization of cyclosiloxanes. In particular, aluminosilicate and bentonite were used as catalysts in the opening of D3 and D4. Such catalysts have proven to be a good choice for the heterogeneous ROP cationic polymerization of cyclosiloxanes (Figure 24). The increase in acid sites due to acid treatment led to the dealumination of materials, which made possible the polymerization of cyclosiloxanes. The structural change in the material caused by the loss of aluminum created the necessary chemical conditions to facilitate the polymerization process. The catalysts were obtained by a relatively simple and economical procedure and were easily separated from the reaction medium. However, product yields were extremely low [127].

**Figure 25:**
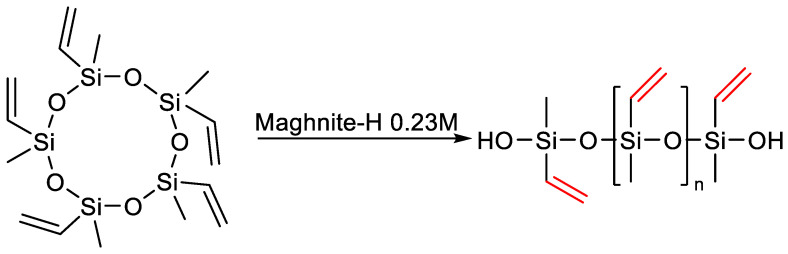
Polymerization of V_5_D_5_ by Maghnite-H^+^.

In recent years, biocatalysis has become increasingly popular; that is, the use of natural catalysts such as clays in an organic synthesis reaction [128,129]. Djamal Eddine Kherroub and co-authors have developed and implemented an alternative method for the synthesis of silicone polymers. This method involves the use of Magnet-H^+^, an aluminosilicate ecocatalyst designed to initiate the polymerization reaction of pentavinylpentamethylcyclopentasiloxane (V_5_D_5_) (Figure 25). A total of 0.1 g of Maghnite-H^+^ was heated under vacuum with mechanical agitation for 30 min before to use. The polymerization was carried out in bulk. The dried amount of Maghnite-H+ was added to a flask containing 5 g of V_5_D_5_, the flask was carried out in an oil bath at 60 °C under reflux with stirring. After 6 h the reaction was stopped by deactivating Maghnite-H^+^ by adding cold water to the reaction mixture. However, the presence of additional steps makes the process more complex and less preferable compared to using standard catalysts [130].

#### 2.2.2. Obtaining Siloxane Telechelics by CROP

The CROP mechanism is used to obtain functional PDMS telechelics widely used in industry: hydride [131,132,133], mercaptopropyl [134], vinyl [135], as well as hydroxybutyl [136], carboxyl [137], and hydroxyl [138].

Today, PDMS-telechelics with “standard” functional groups are obtained for the purpose of their further modification [10]. Thus, Gorodov et al. obtained a series of hydride-containing polymers and copolymers by cationic polymerization of octamethylcyclotetrasiloxane with 1,1,3,3-tetramethyldisiloxane or polymethylhydrosiloxane and hexamethyldisiloxane. The process was carried out at various ratios of reagents in the presence of sulfonic acid resin for 8–10 h at 70 °C. Subsequently, siloxane copolymers containing fragments of undecylenic acid and its esters were synthesized by adding a trimethylsilyl group or tert-butyl undecenoate to the silicon hydride groups of polydimethylmethylhydrosiloxanes by hydrosilylation [139]. In addition, Gorodov V.V. et al. [140] in their review considered the preparation of hydride-containing PDMS and their subsequent functionalization to obtain carboxyl-containing PDMS.

According to the same principle with the same catalyst, polydimethylsiloxanes with side functional hydrosilanes were obtained by Drozdov F.V. et al. [141]. Based on polydimethylsiloxanes (PDMS) with terminal dimethylhydrosilyl or distributed methylhydrosilyl groups in the polymer chain and methyl esters of boronic or phenylboronic acid, cross-linked polyborosiloxanes were obtained by the Pierce–Rubinstein reaction (PBS). Depending on the number and location of methylhydrosilyl groups in the initial PDMS, as well as on the functionality of the boron component, PBS with different macromolecular structures and crosslinking densities were obtained.

In the work of Tasic et al., a cation exchange resin based on macroporous sulfonated cross-linked polystyrene was used as a heterogeneous catalyst for the synthesis of PDMS-telechelics with trimethyl-, hydrido-, vinyl-, and carboxypropyl end groups [142]. In all cases, polymers with a low molecular weight (2500) were obtained, so that later they could be used for the synthesis of block copolymers. Syntheses were performed starting from D4, while the disiloxane co-reagents for inclusion of the functional group were hexamethyldisiloxane (HMDS), 1,1,3,3-tetramethyldisiloxane (TMDS), 1,3-divinyltetramethyldisiloxane (DVTMDS), 1,3- bis(3-carboxypropyl)tetramethyldisiloxane (DCPTMDS).

**Figure 26:**
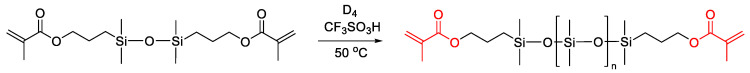
Synthesis of α, ω-bis(methacryl) PDMS.

Benjamin T. Cheesmana et al. prepared acrylate PDMS telechelics by the ROP mechanism in the presence of a trifluoromethanesulfonic acid catalyst. 1,3-bis(methacryl)tetramethyldisiloxane obtained by hydrosilylation of allyl methacrylate was used as a blocking agent (Figure 26) [143]. The methacrylate-terminated PDMS macromonomers synthesized in this study have been successfully used to form films by UV-induced crosslinking, and studies of the properties of crosslinked films are the subject of future publications.

**Figure 27:**
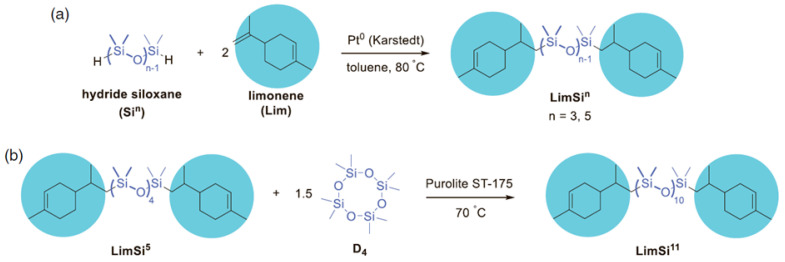
Synthesis of difunctional siloxane derivatives of limonene by hydrosilylation of the corresponding PDMS with functional Si–H groups (**a**) and by the ring-opening polymerization of octamethylcyclotetrasiloxane in the presence of a shorter difunctional siloxane derivative of limonene (**b**) (adapted with permission from Ref. [144]. 2019, Drozdov, F.V.).

In the work of Drozdov F.V. [144], the preparation of limonene functional PDMS by the mechanism of cationic ROP from D4 and difunctional siloxane derivative of limonene in the presence of Purolite ST-175 catalyst was considered (Figure 27). In this work, a series of prepolymers based on difunctional siloxane derivatives of limonene and dithiols with different methylene spacer lengths was obtained by a photoinitiated thiol polyaddition reaction. It has been shown that an increase in both the siloxane and methylene moieties in the starting monomers results in higher molecular weight products (4000–15,000 Da).

**Figure 28:**
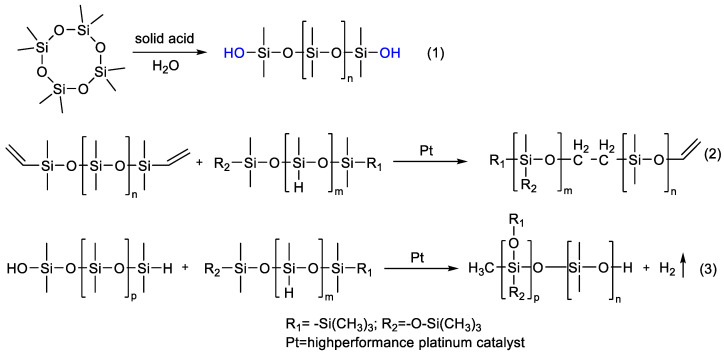
Preparation of hydroxyl-terminated polydimethylsiloxanes (OH–PDMS) and polysiloxane foam (SIF).

Zhang C. et al. [145] report the preparation of hydroxyl-functional PDMS from D4 with water in the presence of solid super acid (Figure 28). An effective method for improving the thermal insulation and stability of polysiloxane foam (SIF) by adjusting the chain length of hydroxyl-terminated polydimethylsiloxane (OH–PDMS) has been described. A series of SIFs were obtained through foaming and crosslinking processes with different crosslinking densities.

### 2.3. Catalytic Rearrangement Reactions for the Preparation of PDMS Copolymers

PDMS with chain-distributed functional moieties play an important role in the chemistry of silicones. These functionalities are not only capable of providing new properties to materials, but are also necessary for further transformations and obtaining new polymers with a given structure and a required set of characteristics.

Among the many chemical processes used in silicone chemistry, there is one universal process that provides a variety of silicones that gives them with unique adaptability to the most controversial consumer requirements. This process is called equilibration or, as it is often called in Russian literature, catalytic rearrangement. It can be used to obtain telechelics homogeneous in molecular weight and structure, and make it possible to obtain random copolymers. Their composition corresponds to the ratio of the initial reagents, which was obtained from a complex mixture of co-hydrolysis products of complex compositions of chlorosilanes, which differ markedly in their reactivity. In addition, it should be noted that the substituents at the silicon atom strongly affect the rate of polymerization of cyclosiloxanes; as a result, it is difficult to obtain copolymers by polymerizing a mixture of cyclosiloxanes with different groups at the silicon atom. This problem can be solved using mixed dimethylcyclotetrasiloxanes, the synthesis of which is considered in the work Talalaeva E.V. et al. [146]. 

**Figure 29:**
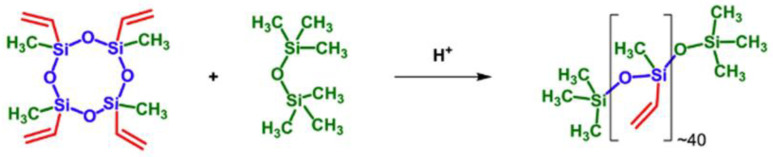
Synthesis of vinyl-containing precursor (/adapted with permission from Ref. [135]. 2018, Temnikov, M.N.).

First of all, it is necessary to note the preparation of functional homopolymers by the catalytic rearrangement reaction. Temnikov M.N. et al. [135] obtained vinyl functional homopolymers from 2,4,6,8-tetramethyl-2,4,6,8-tetravinylcyclotetrasiloxane in the presence of a sulfonic cation exchanger catalyst by the cationic catalytic rearrangement mechanism (Figure 29). The obtained PDMS were functionalized according to the thiol-ene mechanism for the subsequent preparation of aerogels in scCO_2_.

**Figure 30:**
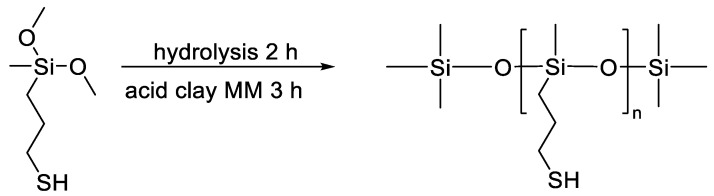
Scheme for obtaining PMMS.

In a study by Cao J. et al. [147], functional mercaptopropyl PDMS was obtained by the mechanism of catalytic rearrangement of (mercaptopropyl)methyldimethoxysilane hydrolyzate in the presence of acid clay (Figure 30). Through the thiol-ene reaction of the obtained PDMS with 2,5,8,11,14,17,20,23-octaoxahexacos-25-ene, a water-soluble comb-shaped polysiloxane was synthesized with a different ratio of polyester and mercaptopropyl groups as side chains.

**Figure 31:**
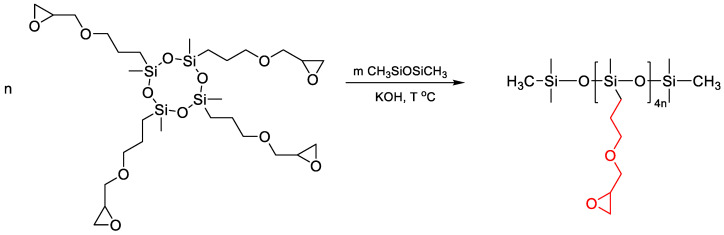
Ring-opening oligomerization of D4^R^ in the presence of potassium hydroxide.

Mukbaniani O. [148] reported on the preparation of epoxy-functional homopolymers from D4 with functional epoxy groups in the presence of an anionic initiator KOH in dry toluene (Figure 31). Further, the reaction of compounds containing epoxy groups with primary and secondary amines was carried out, and the corresponding compounds containing aminohydroxyl groups were obtained. Similarly, a linear methylsiloxane oligomer with regular arrangement of propyl acetoacetate groups in the side chain has been obtained [149]. In this work, the possibility of revealing epoxy groups is open to question; however, the authors confirm the composition and structure of the obtained compounds according to elemental analysis, such as FTIR, and ^1^H, ^13^C, and ^29^Si NMR spectrum. In addition, some properties of linear epoxides have been investigated. According to the given data, the percentage content of epoxy groups in the obtained oligomers is close to that calculated.

**Figure 32:**
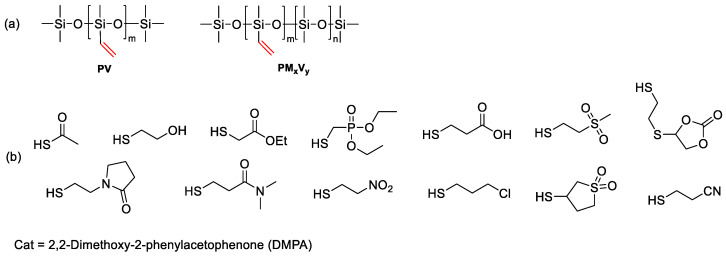
Structures of the Starting Polysiloxanes PV and PM_x_V_y_ (**a**) and of Polar Thiols Selected for Their Functionalization (**b**).

A large number of studies are also devoted to the preparation of copolymers by the mechanism of catalytic rearrangement. Sheima Y. et al. [150] considered the preparation of vinyl functional PDMS by the mechanism of anionic catalytic rearrangement. Then the vinyl groups were converted into polar groups of various nature using an efficient one-step post-polymerization modification of thiol-ene addition (Figure 32). The obtained set of materials was used to establish relationships between structure and properties, the design of side groups, and thermal and dielectric properties. Polymers with a high dielectric constant are promising for creating next-generation energy converters and storage devices with increased energy density.

**Figure 33:**
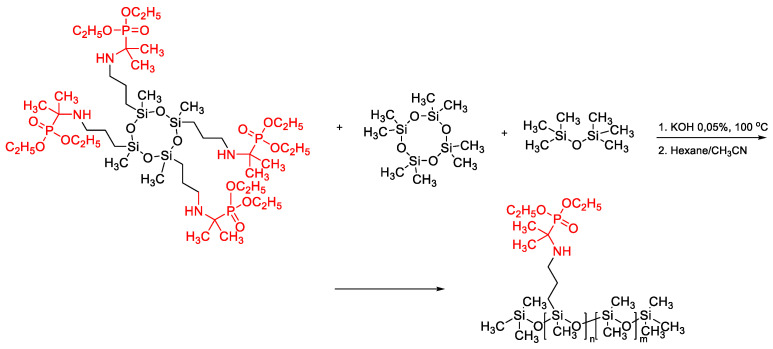
The copolymerization of D_4_ with phosphorus-substituted cyclosiloxane.

Besides to the usual functionalities of PDMS, which are considered everywhere, the introduction of new functional groups cannot be ignored. Sergey A. Milenin et al. used an anionic ROP mechanism to synthesize copolymers based on polydimethylsiloxanes and aminophosphonates from D4, a preformed cyclic siloxane with aminophosphonate functions, and hexamethyldisiloxane in a ratio of 6:0.25:1 in the presence of 0.05 wt.% crystalline KOH was carried out at 100 °C (Figure 33) [151]. Thus, the synthesized cyclosiloxane with phosphorus-containing substituents at silicon atoms and the product of its copolymerization with octamethyltetrasiloxane are promising modifiers for formulations based on polydimethylsiloxanes. Such additives affect the rheology, suppress crystallization, and promote the formation of a cross-linked structure during the thermal-oxidative degradation of polydimethylsiloxanes.

**Figure 34:**
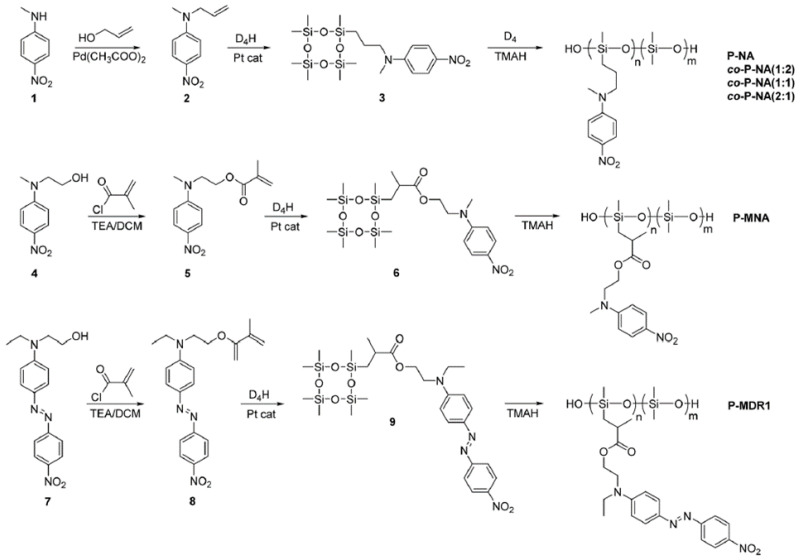
Synthesis of cyclosiloxanes containing nitroaniline and Disperse Red 1 group sand their ring opening polymerization (reprinted with permission from Ref. [152]. 2018, Perju, E).

Perju E. [152] presents the synthesis of three new tetracyclosiloxane monomers modified with either nitroaniline (NA) or the Disperse Red 1 (DR1) group and their ring-opening polymerization reaction in the presence of tetramethylammonium hydroxide (Figure 34). Because of their high dielectric constant of 17.3 at RT. and rather low Tg, NA-modified polymers are attractive as active dielectric materials in actuating elements, capacitors, and flexible electronics [153].

**Figure 35:**
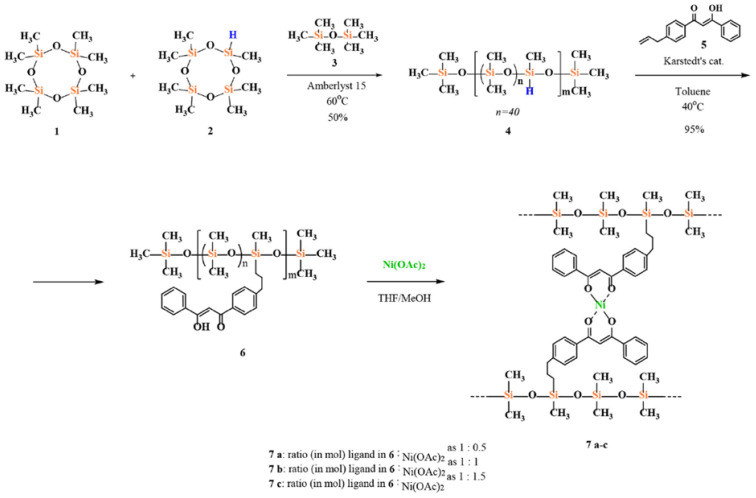
Preparation of cross-linked polymers based on polysiloxane and metal β-diketonate precursors (adapted with permission from Ref. [154], 2021, Kim, E.E.).

Kim E.E. et al. [154] prepared hydride-containing copolymers from D4 and D4^H^ in the presence of Amberlyst 15 catalyst (Figure 35). The resulting copolymers were used to obtain polysiloxanes with distributed dibenzoylmethane groups, on the basis of which a number of new cross-linked polymers were successfully obtained by the interaction of polyligand PDMS with grafted fragments of β-diketonate and nickel (II) acetate.

Morariu S. et al. [80] obtained poly(dimethylsiloxane-co-diphenylsiloxane) by ring-opening anionic copolymerization of D4 and Ph4 using tetramethylammonium hydroxide as a catalyst and a DMF Lewis base as a promoter. Basic catalysts are recommended for opening the octaphenyltetrasiloxane ring. The transition catalyst TMAH was chosen in this work because it can be easily removed at the end of the reaction by thermal decomposition to form volatile compounds (trimethylamine and methanol). DMF was added to increase the reaction rate by complexing the counterion and preventing ion pair formation. Unfortunately, the work does not pay sufficient attention to confirming the structure of the obtained copolymer.

**Figure 36:**
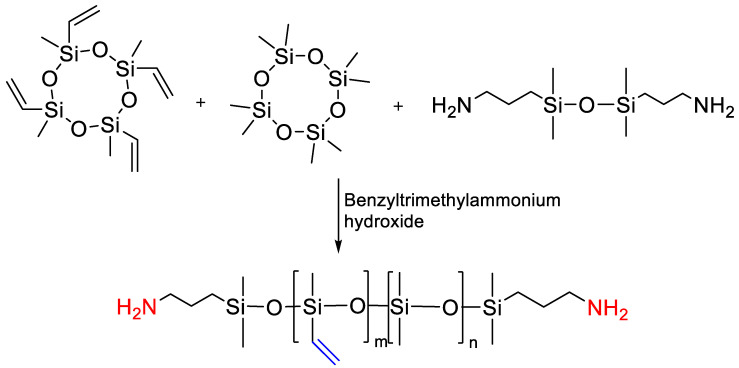
Synthesis of siloxane polymers having terminal amine functionality.

One cannot leave aside a number of works where PDMS copolymers with several types of functionalities are obtained by the catalytic rearrangement mechanism. Among these works, attention is drawn to the work of Bodkhe R.B. [155], where 3-aminopropyl-terminated polydimethylvinyl siloxane (APT-PDMVS) was obtained in the presence of a benzyltrimethylammonium hydroxide catalyst (Figure 36). D4^Vi^ is more highly reactive with base than D_4_, and therefore, to study the reproducibility of the reaction for a given molecular weight, APT-PDMVS equilibration using different chain length end blockers was studied. To this end, a series of six different APT-PDMVS polymers was synthesized using each end blocker, having a target number average molecular weight (M_n_) of 5000, 10,000, 15,000, 20,000, 30,000, and 40,000 g/mol. The mole ratio of D4 to D4^Vi^ was kept constant at 1:1. A number of aminopropyl terminated PDMS polymers having orthogonal carboxylic acid groups have been successfully incorporated into the polyurethane coating system. Further fine tuning of the composition of the acid-functional siloxane polymer and the amount of siloxane included in the polyurethane coating can lead to improved performance of the composite coating.

A number of works are devoted to the synthesis of copolymers with phenyl and vinyl substituents. One of them is the study by Sheima Y. [150], in which homopolymers of poly(methylvinylsiloxane) (PV) and copolymers of poly(dimethyl-co-methylvinyl)siloxanes (PM_x_V_y_) with ratios x/y = 2:8, 4:6, 6:4, and 8:2 were obtained by ring-opening anionic polymerization of the corresponding D4 and D4^Vi^ monomers in the presence of hexamethyldisiloxane as the end blocking agent. The vinyl groups were then converted into polar groups of various natures using an efficient one-step post-polymerization modification by the thiol-ene addition mechanism. In this paper, it is worth noting that the authors make a complex equilibrium system based on simple methods, while maintaining the level of control.

**Figure 37:**
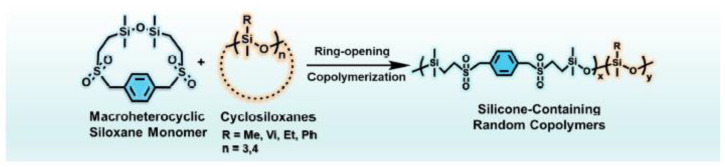
Ring-opening copolymerization of Macroheterocyclosiloxane and Cyclosiloxane (adapted with permission from Ref. [156]. 2021, Guo, M.).

Guo M. et al. [156] investigated the ring-opening copolymerization (ROCP) of benzylsulfonyl macroheterocyclosiloxane (BSM) and five different cyclosiloxanes (Figure 37). Here, a general approach was developed for the synthesis of benzylsulfonyl-containing silicone copolymers with various substituents, including methyl, vinyl, ethyl, and phenyl. A range of variable inclusion copolymers (6% to 82%) BSM was made by varying the comonomer ratio and using KOH as a catalyst in a mixture of dimethylformamide and toluene as solvents. The resulting copolymers exhibit different composition-dependent properties and unique viscoelasticity. Notably, the surface and fluorescent characteristics, as well as the glass transition temperatures of the copolymers, can be adapted by changing the amount of BSM. Unlike typical sulfone-containing polymers such as poly(olefin sulfones), the resulting copolymers exhibited excellent thermal and hydrolytic stability. The universal strategy developed in this study provides a platform for the development of innovative silicone copolymers with controlled structure and performance.

**Figure 38:**
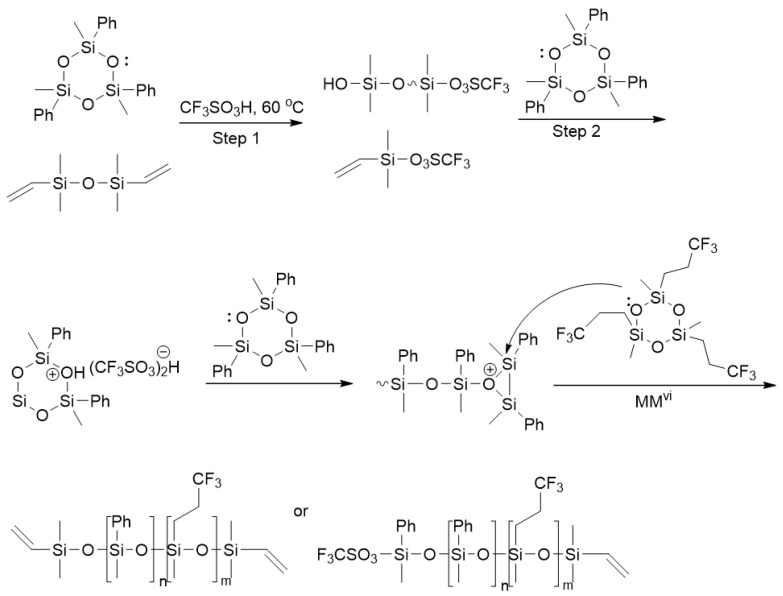
Possible mechanism of copolymerization of D_3_^F^ and D_3_^Ph^ initiated by CF_3_SO_3_H (adapted with permission from Ref. [157]. 2016. Fei, H.F.).

Fei H.F. et al. [157] obtained trifluoropropylmethylsiloxane–phenylmethylsiloxane gradient copolysiloxanes by copolymerization of 1,3,5-tris(trifluoropropylmethyl)cyclotrisiloxane (D3^F^) and phenylmethylcyclotrisiloxane (DPhrisiloxane) (Figure 38). An analysis of the reactivity ratios showed that the reactivity of D3^F^ with respect to anionic ROP is higher than that of D3^Ph^; however, D_3_^F^ showed lower reactivity compared to D3^Ph^ during cationic ROP. Gradient copolymers of type AB and BAB were obtained due to the difference in the reactivity of the monomers. The microstructure of the copolymers was characterized by ^29^Si NMR spectroscopy, gel permeation chromatography, and differential scanning calorimetry.

**Figure 39:**
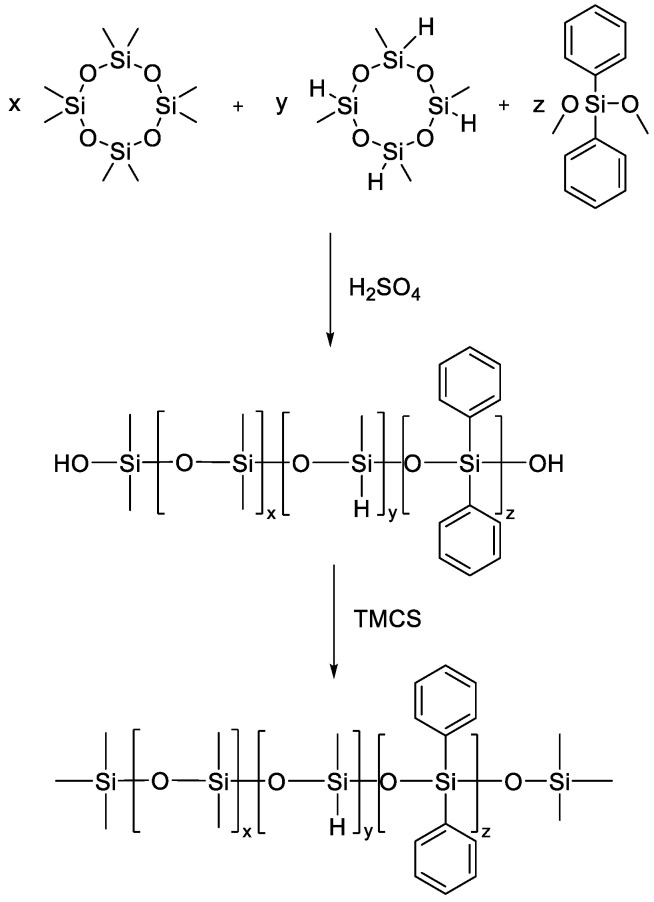
Synthesis of V-PDMPS.

The work of Indulekha K. [158] shows the Synthesis of trimethylsilyl terminated poly(dimethyl-co-methylhydrogen-codiphenyl)siloxane (TMS–PDMHS). TMS–PDMHS was synthesized by the cationic ring opening polymerization of cyclic siloxanes, D4 and D4^H^ in presence of DPDMS using H_2_SO_4_ as catalyst (Figure 39). The structure of the obtained copolymers was confirmed by ^1^H, ^29^Si NMR data. The resulting compound was further used as a crosslinking agent.

**Figure 40:**
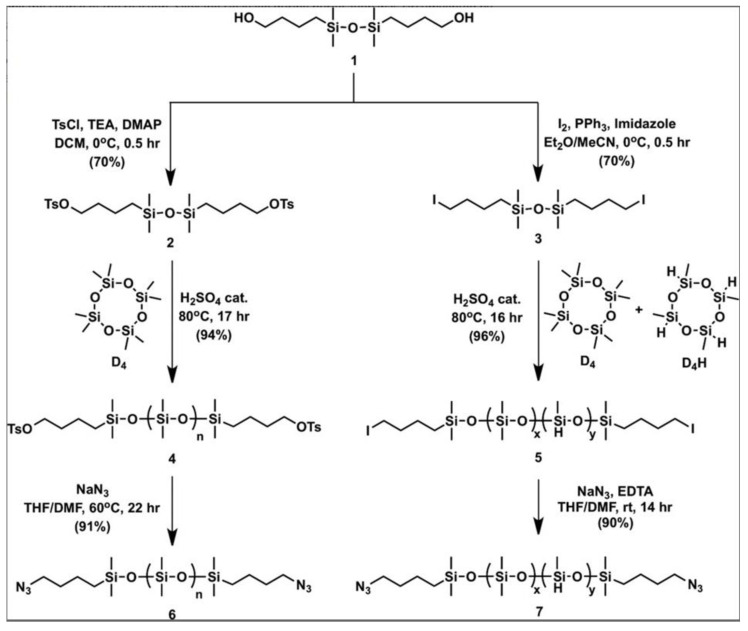
Synthesis of bis-azidepoly(siloxane) B-blocks and via cationic ring-opening polymerization of cyclic siloxane monomers using the end-blocker method (adapted with permission from Ref. [159]. 2012, Isaacman, M.J.).

Isaacman M.J. et al. [159] synthesized bifunctional polysiloxanes with tosylate functional groups, carried out by cationic polymerization of D4 in the presence of sulfuric acid. Similarly, copolymers with iodide functional end groups were obtained using D4 and D4^H^ as monomers (Figure 40). The compounds, which was obtained by converting iodide or tosylate end groups into azides, were converted into interactive partners for alkyne-functionalized poly(oxazoline) A-blocks.

**Figure 41:**
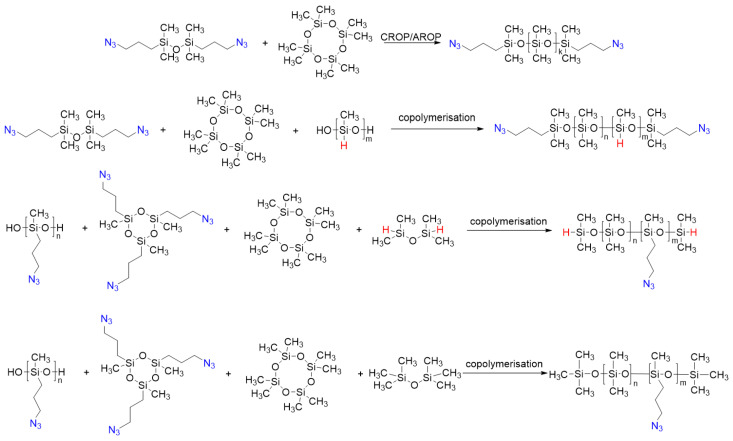
Synthesis of polydimethylsiloxanes (PDMS) with azidopropyl functional groups at the silicon atom by ring-opening polymerization (ROP).

It is important to note that the ROP and catalytic rearrangement reactions have not only made it possible to obtain PDMS with well-known functionalities for many decades, but also open up opportunities for introducing new functions that are relevant in our time. Thus, in the work of Milenin S.A. et al., for the first time the whole variety of options for the synthesis of polydimethylsiloxanes (PDMS) with azidopropyl functional groups at the silicon atom was demonstrated by classical ring-opening polymerization (ROP) and catalytic rearrangement of siloxanes in the presence of a strong acid (CF_3_SO_3_H) (Figure 41) [160]. The proposed method was used to obtain not only PDMS containing azidopropyl functional groups at both ends of the polymer chain (telechelic), but also PDMS with an irregular structure containing various proportions (5–50%) of azidopropyl functional groups in the main polymer chain. It is important that the proposed method also turned out to be effective for the synthesis of PDMS containing both azidopropyl and hydridosilyl functional groups. As a result, PDMS were obtained with different mutual arrangement of two types of functional groups in the PDMS chain. The used method of catalytic rearrangement of low molecular weight siloxanes made it possible to obtain PDMS with azidopropyl functional groups in a wide range of molecular weights from 2000 to 88,000 according to gel permeation chromatography (GPC). In addition, the possibility of further modification of the resulting azidopropyl-functional PDMS, as well as multifunctional PDMS containing azidopropyl and hydridosilyl functional groups simultaneously, by azide-alkyne cycloaddition reactions was demonstrated.

## 3. Conclusions

As we have seen, the ROP method as applied to functional linear oligomers is being actively developed and remains the main method for obtaining PDMS oligomers with a wide range of functional endings. There are many examples of reproducing this method to obtain compounds with given molecular parameters and the type of functional groups in the literature. However, it is quite obvious that the use of new catalysts, substrates based on natural compounds, and the improvement of experimental techniques makes it possible to count on the synthesis of new original silicone polymers, opening up new areas of their practical application.

Siloxane monochelics with polymerizable groups (macromonomers) made it possible to achieve a breakthrough in the properties of polymeric materials by changing the nature of intermolecular interactions in networks of molecular brushes. It is possible to obtain materials with both very low and very high mechanical modulus in these systems, in which siloxanes play a dominant role. The level of regulation of properties is amazing; it is not by chance that the authors call this approach the polymer genome. It would be impossible to make this breakthrough without the synthesis of mono- and telechelics with strictly specified molecular parameters. Therefore, the emergence of new libraries of mono- and telechelics based on PDMS containing azide and acetylene functions, opening up a rich chemistry of azide-alkyne cycloaddition, promises the emergence of new polymer systems and materials with an unusual set of properties and the possibility of their fine tuning. All of this will make it possible to control the properties and functions of new polymers and materials based on them through fine control of the structure.

Thus, the growing choice of functional endings both in mono- and telechelics, and in polyfunctional linear matrices with a controlled frequency of distribution of functional groups, as well as the quality of these functionalities, which allow the formation of target structures based on atom-saving addition processes, indicate a qualitative leap in design of silicone structures of complex architecture. In turn, the unique properties of materials based on such polymers are an effective stimulus for the further development of old, well-proven methods for the synthesis of polymers, which undoubtedly include ring-opening and equilibration reactions.

## Data Availability

Not applicable.
